# Cincinnati Medical Society

**Published:** 1833

**Authors:** 


### ART. XI. CINCINNATI MEDICAL SOCIETY.

This is a society of emulation, instituted on the 4th of March, 1831, and incorporated during the last session of the Legislature. It embraces a majority of the physicians of the city, and holds weekly meetings for lectures and debates. About two months ago, it resolved on creating three new means of improvement, which are of sufficient general interest, to justify our stating them to the profession.

1. An *Herbarium*, especially of the medicinal plants of the West. Dr. Colby has been appointed its Curator, and, as we are authorized to say, will be happy to open a correspondence and carry on exchanges of specimens, with any western physicians who may choose to direct their attention to our native plants. Those who are not good practical botanists, from the want of the requisite books, would find it to their advantage, to send specimens to that gentleman; numbering them and keeping duplicates with the same numbers, in their own possession, which might be labelled according to the answers he should give. The society, moreover, would thankfully receive specimens of all kinds, as it is anxious to render its Herbarium, of immediate value to the profession.

2. A *Cabinet* of *Pharmacy*, which shall embrace, along with all kinds of pharmaceutic preparations, specimens of the various minerals belonging to our own and other countries, which are connected with the Materia Medica. Dr. Hermange has been appointed Curator of the Cabinet; and being a skillful practical chemist, will with pleasure, analyze any minerals of the West, that may be transmitted to him for that purpose.

3. A *Library*. A considerable sum of money has been contributed by members of the society, for this important object; and they have already presented more than 100 volumes as a foundation. Several gentlemen, not members of the society, as the medical professors of Transylvania University, and some who are not even physicians, have lent a helping hand, to this laudable undertaking; which is designed to embrace a number of periodical works—European as well as American. We trust that authors and publishers, who may see this notice, will be inclined to send copies of their works to the library.

The officers of the Society are—

DR. RIVES, *President*,
DR. HENRY, *Vice President*,
MR. WOOD, *Chairman*,
DR. SHOTWELL, *Treasurer*,
MR. BONNER, *Secretary*,
MR. DODGE, *Librarian*,
DR. HERMANGE, *Curator of the Cabinet*,
DR. COLBY, *Curator of the Herbarium*.

